# Using R in Taverna: RShell v1.2

**DOI:** 10.1186/1756-0500-2-138

**Published:** 2009-07-16

**Authors:** Ingo Wassink, Han Rauwerda, Pieter BT Neerincx, Paul E van der Vet, Timo M Breit, Jack AM Leunissen, Anton Nijholt

**Affiliations:** 1 Human Media Interaction Group, University of Twente, Enschede, The Netherlands; 2 MicroArray Department & Integrative Bioinformatics Unit, University of Amsterdam, Amsterdam, The Netherlands; 3 Laboratory of Bioinformatics, Wageningen University, Wageningen, The Netherlands

## Abstract

**Background:**

R is the statistical language commonly used by many life scientists in (omics) data analysis. At the same time, these complex analyses benefit from a workflow approach, such as used by the open source workflow management system Taverna. However, Taverna had limited support for R, because it supported just a few data types and only a single output. Also, there was no support for graphical output and persistent sessions. Altogether this made using R in Taverna impractical.

**Findings:**

We have developed an R plugin for Taverna: RShell, which provides R functionality within workflows designed in Taverna. In order to fully support the R language, our RShell plugin directly uses the R interpreter. The RShell plugin consists of a Taverna processor for R scripts and an RShell Session Manager that communicates with the R server. We made the RShell processor highly configurable allowing the user to define multiple inputs and outputs. Also, various data types are supported, such as strings, numeric data and images. To limit data transport between multiple RShell processors, the RShell plugin also supports persistent sessions. Here, we will describe the architecture of RShell and the new features that are introduced in version 1.2, i.e.: i) Support for R up to and including R version 2.9; ii) Support for persistent sessions to limit data transfer; iii) Support for vector graphics output through PDF; iv)Syntax highlighting of the R code; v) Improved usability through fewer port types.

Our new RShell processor is backwards compatible with workflows that use older versions of the RShell processor. We demonstrate the value of the RShell processor by a use-case workflow that maps oligonucleotide probes designed with DNA sequence information from Vega onto the Ensembl genome assembly.

**Conclusion:**

Our RShell plugin enables Taverna users to employ R scripts within their workflows in a highly configurable way.

## Background

The open source workflow system Taverna [1] provides access to and integration of many life science web services. However, not all desired data-analysis procedures are available as web services. Therefore, support for scripting is essential and by default, Taverna provides scripting in Java. Many scientists are not familiar with Java and prefer other languages, such as R, a statistical language commonly used by many life scientists [2]. Taverna had limited support for R. We developed an R plugin for Taverna: RShell, which removes the limitations of R support in Taverna workflows. RShell can be incorporated in a Taverna workflow as a processor for executing R scripts. Although RShell was already sketchily introduced in Li et al. [3], here we present a detailed description of the RShell functionality and architecture. This was also motivated by several requests from Taverna and R users. Additionally, we introduce new functionality of our current RShell version 1.2.

## Implementation

**Figure 1:**
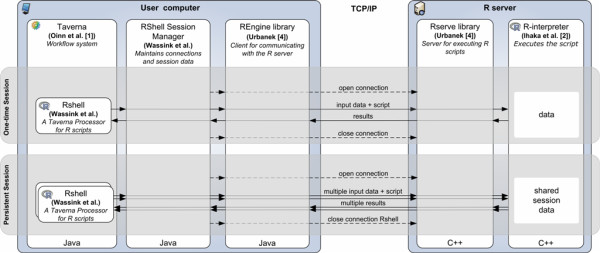
**The design of RShell**. Each RShell processor communicates with the R-interpreter through the RShell Session Manager. The RShell Session Manager sets up and maintains the connection with the R interpreter with the RServe library installed through the REngine java library. This figure is adapted from Li et al [3]. images/RShell.

RShell is based on a client-server architecture. It requires a local or remote installation of the R-interpreter with the Rserve library [4] installed. The Rserve library turns the R-interpreter into a server, which enables other applications to communicate with the R-interpreter by means of a socket connection. From here on, the R-interpreter with the Rserve library installed will be denoted as the *R server*. RShell uses the Java library named REngine to establish and maintain connection between Taverna and R server. To execute R scripts, the RShell processor sends the script and the input data via the RShell session manager to the R server, which delegates the script to the R-interpreter and sends the results back to RShell (Figure 1). This new version is fully compatible up to and including R 2.9.

### Configuring the RShell processor

The RShell processor is highly configurable: the user can define the script to be executed, the input ports to feed the relevant data, and the output ports to extract the created data. The user can configure the RShell processor by invocating the RShell processor in Taverna's "advanced model explorer". This will show a configuration dialog containing four tabs for the script, the input ports, the output ports and the connection settings.

Syntax highlighting is available in the scripting tab, to help the user write correct R code. The keywords are highlighted in blue; the inputs and outputs of the RShell are highlighted in pink.

RShell supports multiple input and output ports. Each port has a name, corresponding to the variable name it represents, as well as a type, corresponding to the type of data it holds. RShell uses the data type to determine how to exchange data with Taverna. RShell supports booleans, numeric values (both integers and floating point numbers), strings, and, vector of numerics and strings. Inputs and outputs of these data types can directly be used as variables in the R script. RShell provides the text-file data type to handle large data. Ports of this type can be read and written as normal R tables. The PNG and, since version 1.2, the PDF data type are available for graphical output. PNG can be used bitmap graphics; PDF for vector graphics. PNG and PDF outputs are handled as graphics devices in the standard R fashion. Input ports and output ports can be defined using the input ports and output ports tab.

RShell can execute any R script as long as the required libraries are installed in the R server. By default, the RShell processor is configured to use a locally installed R server serving at address *localhost*, port *6311*. It can be configured to use a remote installation of R instead, using the connections tab. This can be useful when, the user is not able to install R, a central installation of R with a specific set of installed libraries is used, or R is installed in a grid environment. Multiple R servers can be accessed within the same workflow.

### Persistent sessions

RShell supports persistent sessions to prevent unnecessary data transfer. The user can enable persistent sessions in the connection tab. When these are enabled, all input data, output data and script variables will be kept in memory of the R-interpreter until the whole workflow execution is finished. Multiple RShell processors in the same workflow are able to use the data provided by previously used processors, without requiring data links between these processors, as long as they use the same R server, Although persistent sessions can be very useful, they have some limitations: i) sessions can only be used among RShell processors, ii) RShell processors involved in a single persistent session have to access the same R server (but RShells devoted to a different session can access other R servers), and iii) it takes extra effort to keep a provenance log of data kept at the R interpreter. Taverna manages all data consumed and produced by processors. By using persistent sessions, Taverna is not aware of the data generated by one RShell processor and used by another RShell processor. If the user wants to record data generated by an RShell processor, he/she can do that by defining an output port for that variable.

## Use-case: mapping oligonucleotides to a genome DNA-sequence

**Figure 2:**
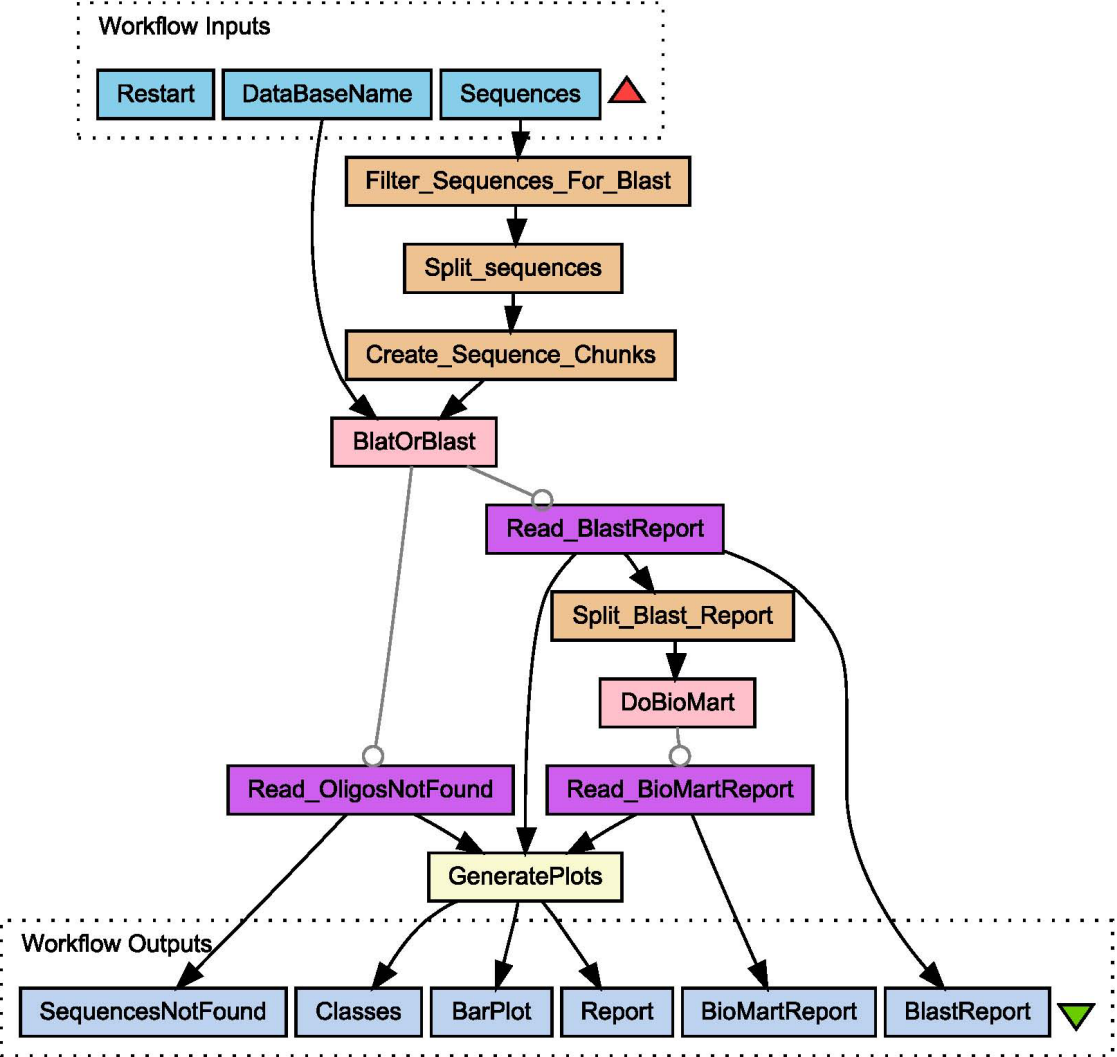
**The use case workflow**. Backbone of the workflow that maps oligonucleotides to an assembly. The RShell processor is the final task in the workflow, used to analyse the oligo mapping. images/DiagramAbstract.

**Figure 3:**
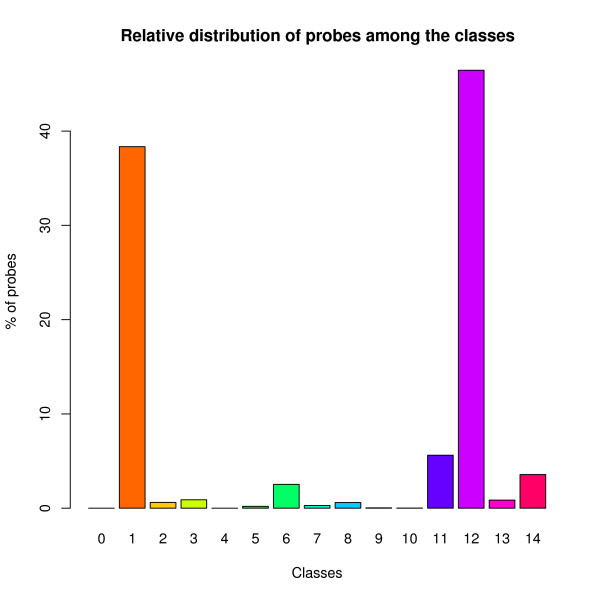
**Plot of the use case results**. Number of probes per class. images/BarPlot.

We used RShell in the design process of a zebrafish microarray (Figure 2) [see Additional file 1 for complete workflow]. A microarray with 15k probes of 60-mer oligonucleotides has been designed on gene sequences from Vega  and Ensembl  that are also known in the Zebrafish Information Network  of the genome DNA-sequence assemblies. For zebrafish, the VEGA set is not a subset of the Ensembl set. To judge the agreement that exists between the different assembly annotations, we mapped the Vega-designed probes onto the Ensembl assembly in the following way. All probe sequences are aligned to the Ensembl assembly. Hits with an e-value below 1.5e-4 are considered to be able to contribute to the hybridization signal on the microarray [5]. Next, for each hit, a query is performed to check which genes and/or transcripts are present at the hit location in the genome. Finally, each probe with at least one hit is further classified [see Additional file 2] based on the number of hits, genes and transcripts. For this classification, an additional lower cut-off is applied (e-value below 1e-12) and the possibility of the occurrence of intron-spanning probes has been considered. Therefore, probe sequences that show two or more hits located close together on the genome and that constitute a continuous stretch of more than 57 nucleotides on the probe, are labeled intron-spanning probes. For the alignment, BioMoby Blat and Blast services at WUR  are used. Gene and transcript finding is performed by the Ensemble 51 Genes BioMart service . The RShell processor is used to implement the classification and the visualisation in the workflow. In Figure 3 [see Additional file 2], one can see that 38.3% of the Vega designed probes have a single hit on the Ensembl assembly (class 1), whereas 3.0% are linked to multiple transcripts of a single gene (class 5–7). 5.6% of the probes are hitting multiple Ensembl genes with an e-value below 1e-12 (class 11). For these genes the Ensembl and Vega annotations clearly disagree. For 46.5% of the probes no transcript is found although they have a hit on the genome assembly below 1e-12 (class 12). These probes target Vega transcripts that are absent in the Ensembl annotation. With RShell, we have been able to implement the use-case as a workflow including the statistical analysis. The complete workflow and the input data as well as a test set can be downloaded from . The workflow was run on a 3 GHz Linux PC with 1.5 GB internal memory and took approximately six hours to complete.

## Conclusion

With the introduction of our RShell plugin, scripting in R is now available for Taverna. RShell has a configurable processor that is able to execute any R script that can be executed by the available installation of the R-interpreter. The client-server architecture enables a centralised installation of the R-interpreter containing all frequently used libraries used by an organisation. The support for persistent sessions in RShell 1.2 helps to prevent data transfer overload.

RShell 1.0 comes with the standard installation of Taverna. RShell 1.2 can be downloaded as a plugin for Taverna and contains several improvements, such as support for vector graphics and persistent sessions

## Availability and requirements

**Project name: **RShell v1.2

**Project home page: **

**Operating system(s): **Any (Java)

**Programming language: **Java

**Other requirements: **Java 1.6, Taverna 1.3+ and R with the Rserve library installed. Taverna, R and Rserve are all open source and freely available.

**Licence: **GNU GPL

## Competing interests

The authors declare that they have no competing interests.

## Authors' contributions

IW has implemented the RShell processor for Taverna. HR has designed the use case to demonstrate the RShell processor. PN has implemented the Blast and Blat service used in the use-case. IW and HR have implemented the workflow.

The authors wish it to be known that, in their opinion, IW, HR and PN should be regarded as joint first authors. All authors have read and approve the final manuscript.

## Supplementary Material

Additional file 1 **Complete workflow**. The expanded version of the workflow designed for the use-case. Click here for file

Additional file 2 **Table of the use case results**. Table representing the description of classes and the number of probes found in each class. Click here for file
