# Supplementary figures and images for: Using R in Taverna: RShell v1.2
Source: BMC Res Notes. 2009 Jul 16;2:138. doi: 10.1186/1756-0500-2-138 (PMC2717104; doi:10.1186/1756-0500-2-138)

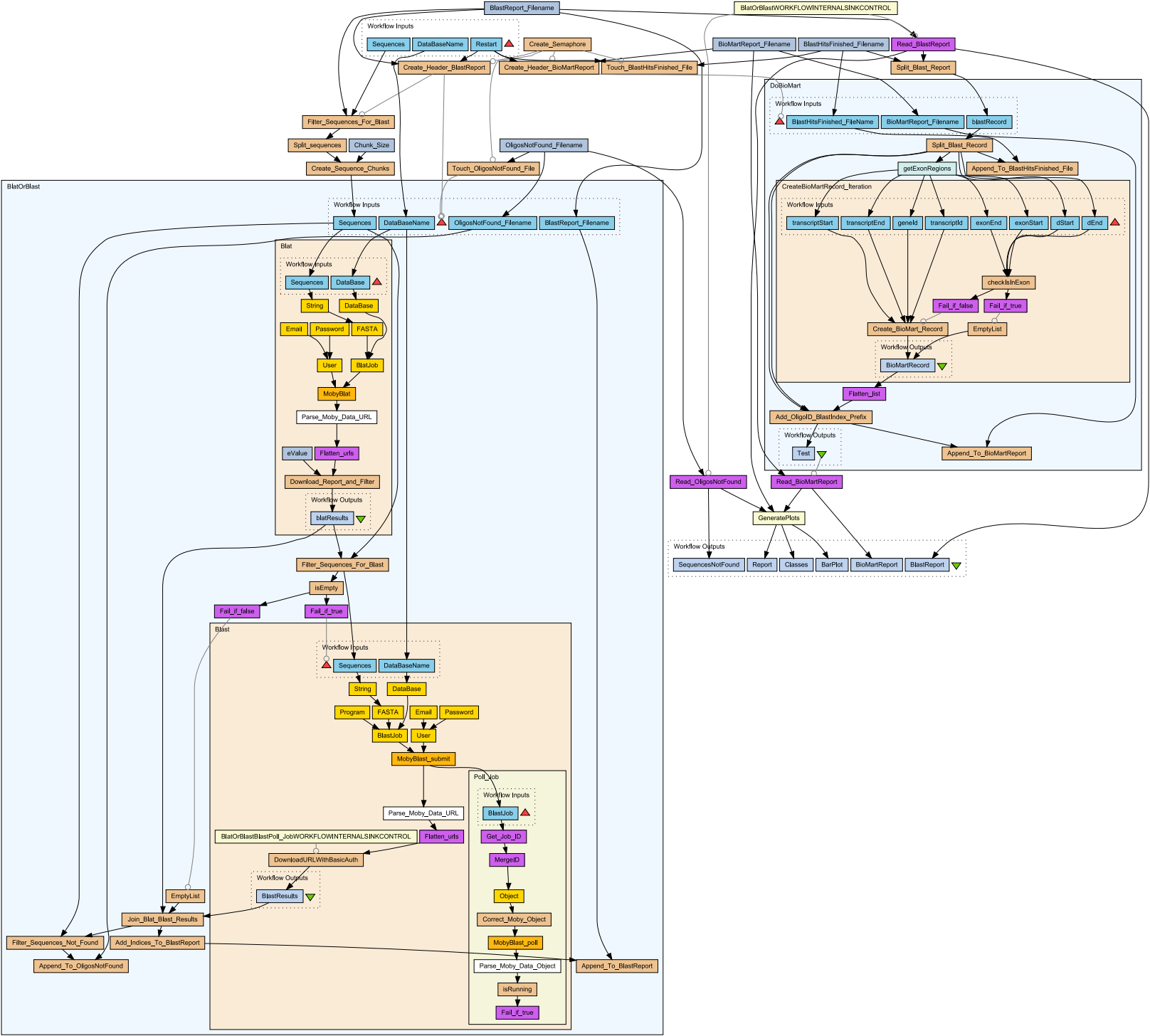

Supplement: Additional file 1 — Complete workflow. The expanded version of the workflow designed for the use-case. [file 1756-0500-2-138-S1.pdf]
