# Supplementary material for: Using R in Taverna: RShell v1.2
Source: BMC Res Notes. 2009 Jul 16;2:138. doi: 10.1186/1756-0500-2-138 (PMC2717104; doi:10.1186/1756-0500-2-138)
Supplement: Additional file 2 — Table of the use case results. Table representing the description of classes and the number of probes found in each class. [file 1756-0500-2-138-S2.pdf]

Table 1: Table of the use case results

Class	Description	e-value <1e-5	e-value <1e-12	# approved transcripts	# approved genes	intron spanning	# probes	% probes
0	no hit	0	0	na	na	na	0	0.0
1	single hit, single transcript, single gene	1	1	1	1	na	3128	38.3
2	multiple hits, single transcript, single gene, intron spanning	>1	0	1	1	+	50	0.6
3	multiple hits, single transcript, single gene, possible intron spanning	>1	>0	1	1	(+) [a]	73	0.9
4	multiple hits, single transcript, single gene, no intron spanning	>1	>0	1	1	-	0	0.0
5	multiple hits, multiple transcripts, single gene, intron spanning	>1	0	>1	1	+	16	0.2
6	multiple hits, multiple transcripts, single gene, possible intron spanning	>1	>1	>1	1	(+) [a]	206	2.5
7	multiple hits, multiple transcripts, single gene, no intron spanning	>1	>1	>1	1	-	23	0.3
8	single hit, does not meet additional criteria [b]	1	0	0	0	na	49	0.6
9	multiple hits, single transcript, do not meet additional criteria [b]	>1	0	1	1	na	3	0.0
10	multiple hits, multiple transcripts, do not meet additional criteria [b]	>1	0	>1	1	na	1	0.0
11	multiple hits, multiple genes	>1	>1	>1	>1	na	458	5.6
12	no transcript found but hit(s) meet additional criteria [b]	>0	>0	0	0	na	3789	46.5
13	no transcript found and hit(s) do not meet additional criteria [b]	>0	0	0	0	na	70	0.9
14	multiple hits, single transcript, single gene plus hit without transcript found and hits meet additional criteria [b]	>1	>1	>0	>0	na	291	3.6

[a] Oligo below e-value cut-off 1e-12, but also intron spanning criteria met.
[b] Additional criteria: either e-value below 1e-12 or intron spanning.
